# Valorization of fishery industry waste: Chitosan extraction and its application in the industry

**DOI:** 10.1016/j.mex.2024.102892

**Published:** 2024-08-05

**Authors:** Maricarmen Iñiguez-Moreno, Berenice Santiesteban-Romero, Elda M. Melchor-Martínez, Roberto Parra-Saldívar, Reyna Berenice González-González

**Affiliations:** a Tecnologico de Monterrey, School of Engineering and Sciences, Monterrey 64849, Mexico; b Tecnologico de Monterrey, Institute of Advanced Materials for Sustainable Manufacturing, Monterrey 64849, Mexico

**Keywords:** Biopolymers, Chitin, Chitosan: Waste valorization, Green extraction

## Abstract

Waste from the fishing industry is disposed of in soils and oceans, causing environmental damage. However, it is also a source of valuable compounds such as chitin. Although chitin is the second most abundant polymer in nature, its use in industry is limited due to the lack of standardized and scalable extraction methods and its poor solubility. The deacetylation process increases its potential applications by enabling the recovery of chitosan, which is soluble in dilute acidic solutions. Chitosan is a polymer of great importance due to its biocompatible and bioactive properties, which include antimicrobial and antioxidant capabilities. Chitin extraction and its deacetylation to obtain chitosan are typically performed using chemical processes that involve large amounts of strongly acidic and alkaline solutions. To reduce the environmental impact of this process, extraction methods based on biotechnological tools, such as fermentation and chitin deacetylase, as well as emerging technologies, have been proposed. These extraction methods have demonstrated the potential to reduce or even avoid using strong solvents and shorten extraction time, thereby reducing costs.

Nevertheless, it is important to address existing gaps in this area, such as the requirements for large-scale implementation and the determination of the stoichiometric ratios for each process. This review highlights the use of biotechnological tools and emerging technologies for chitin extraction and chitosan production. These approaches truly minimize environmental impact, reduce the use of strong solvents, and shorten extraction time. They are a reliable alternative to fishery waste valorization, lowering costs; however, addressing the critical gaps for their large-scale implementation remains challenging.

## Review Highlights

• Biotechnological chitosan extraction is 3-5 times more efficient than chemical methods.
• Incorporating emerging technologies improves the quality and yields of chitosan.
• Modification of chitosan enhances its properties, expanding its application spectrum.

## Graphical abstract

**Figure:**
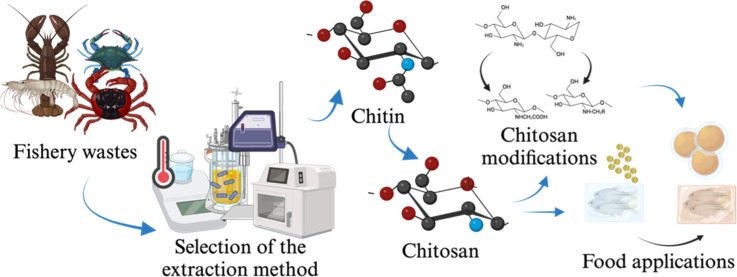

Specifications table

**Table:** 

Subject area:	Agricultural and Biological Sciences
More specific subject area:	Valorization of fishery industry by chitosan extraction
Name of the reviewed methodology:	Chitosan extraction
Keywords:	Biopolymers, chitin, chitosan, waste valorization, green extraction
Resource availability:	Not applicable
Review question:	What is the main extraction process followed for chitin and chitosan extraction from marine sources? What are the biotechnological methods and the main conditions used to obtain chitin and chitosan extraction from marine sources? What is the effect of the combination of emerging technologies and biotechnological tools on chitosan extraction? What are the main chitosan modifications to improve its application in the food industry sector?

## Background

The fishing industry significantly contributes to global waste production by discarding large amounts into terrestrial and aquatic environments, resulting in various environmental issues [1]. Crustacean and fish waste decompose rapidly at warm temperatures through anaerobic decomposition, during which proteins and other nitrogenous compounds are broken down, releasing gasses such as carbon dioxide (CO_2_), methane, amines, diamines, ammonia (NH_3_), and hydrogen sulfide (H_2_S) [[2], [3], [4]]. This process can smother living organisms, promote invasive species on the seabed, and contribute to climate change and rising temperatures [3,5]. The decomposition of organic fishing waste alters the color and odor of the water, promotes the growth of microorganisms, and produces unpleasant odors that contribute to air pollution and public discomfort. These gasses also lower dissolved oxygen levels, adversely affecting aquatic life. When disposed of in landfills, untreated seafood waste increases soil moisture, salinity, electrical conductivity, and inorganic carbon content, which impacts the growth and abundance of prokaryotic organisms in the soil [5]. A life cycle assessment study found that the seafood processing industry contributes to 0.079 kg SO_2_-equivalent acidification, 9.66 kg CO_2_-equivalent climate change, 0.02 kg PO_4_-equivalent eutrophication, 0.17 kg 1,4-DCB-equivalent human toxicity, and 0.0015 kg ethylene-equivalent photochemical oxidation [4,6]. These metrics help understand the environmental burden associated with seafood processing.

Several regulations and policies are in place globally, regionally, and nationally to mitigate the impact of fishery waste disposal. Internationally, the FAO Code of Conduct for Responsible Fisheries and the MARPOL convention Annex V set guidelines and prohibitions on waste disposal from ships. The UN Convention on the Law of the Sea also mandates measures to protect the marine environment from pollution, including fishery waste [7,8]. National regulations, such as the United States Magnuson-Stevens Fishery Conservation and Management Act, Australia's Fisheries Management Act 1991, and Canada's Fisheries Act, provide frameworks for sustainable practices and waste management in fisheries. In addition, local and industry initiatives such as Fishing for Litter programs, eco-labeling by the Marine Stewardship Council, and the Provision of Port reception facilities, further support the reduction and proper disposal of fishery waste, promoting environmentally responsible fishing practices [9].

The fishing industry generates waste, which is relevant for valorization, especially chitin, a biopolymer abundantly found in the exoskeletons of crustaceans and insects [10,11]. Chitin presents challenges in its application due to its inherent insolubility [12]. However, deacetylation can transform it into chitosan, which is soluble in diluted acidic solutions, making it suitable for various industrial uses [13]. Conventional chitin and chitosan extraction uses strong acidic and alkaline solutions for demineralization, deproteinization, and deacetylation [14]. These solvents can be harmful to the environment and human health, for that reason, biotechnological tools like fermentation and the use of emerging technologies such as ultrasound-assisted extraction and microwave irradiation have been proposed to reduce or eliminate the use of those compounds. Even when these processes enable the obtention of high-yield quality polymers, their optimization for large-scale implementation is challenging [[15], [16], [17], [18]]. In addition, chitosan applications in industry can be limited by the low solubility at neutral pH and the low barrier properties compared with synthetic polymers. Several researchers have assessed grafting and copolymerization as alternatives to increasing its application in the food sector to solve these drawbacks and enhance its properties, including solubility [[19], [20], [21]]. The study, optimization, and implementation of these processes at a large scale are of vital importance due to the wide range of chitosan applications. Biodegradability and biocompatibility make it a valuable material in pharmaceuticals and medicine, where it is used in drug delivery systems, wound dressings, and tissue engineering [22,23]. In the food industry, its antimicrobial properties could extend the shelf life of products and ensure food safety. Chitosan also plays a crucial role in water treatment by purifying water and removing contaminants [24]. In agriculture, this polymer enhances plant growth and protects crops from pathogens, while in cosmetics, it is valued for its moisturizing and wound-healing effects [[25], [26], [27]]. Furthermore, its potential to develop biodegradable films and packaging offers an eco-friendly alternative to conventional plastics [22,28,29]. For these reasons, this review aims to show the wide range of conditions used for the extraction process of chitin by chemical method and demonstrate the advantages and gaps in knowledge in implementing biotechnological tools and emerging technologies for this purpose. Furthermore, it explores alternatives for performing chitosan modification, discussing common techniques and applications across various sectors such as food and medicine.

## Method details

**Fig. 1:**
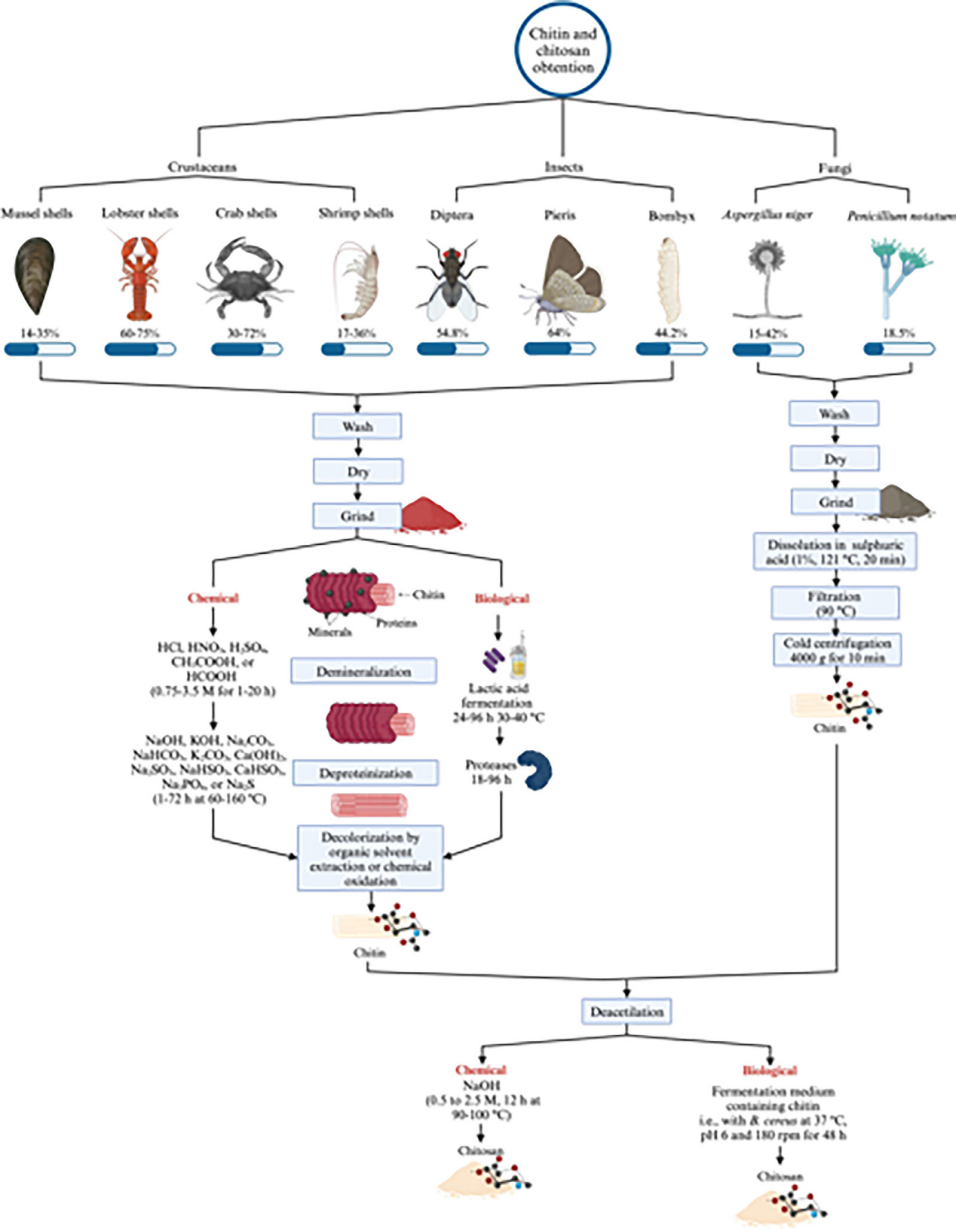
Main natural chitin sources and common extraction processes.

Fishing activities are known for producing a substantial amount of waste including bones, shells, heads, skins, and visceral parts, which are usually disposed of after the consumption of the flesh [30]. It is estimated that in 2020, 36.33 million tons of crustaceans and mollusks were produced [31], generating around 18 million tons of waste per year, which includes crab, shrimp, lobster, mussel, oyster, and clam shells [32]. Most of them, if converted, are transformed into low-value products such as fertilizers and animal feed [12]. Even though it is well known that these wastes contain high-value components, like proteins, vitamins, pigments, amino acids, and polysaccharides like chitin, those can be revalorized and introduced back into industries with enhanced worth [18,33]. Chitin, the second most abundant biopolymer, is a polysaccharide consisting of poly β-(1→4)-N-acetyl-D-glucosamine mainly produced from industrial waste [34]. This biopolymer can be obtained from different sources, such as the exoskeletons of marine organisms like crustaceans (e.g., lobster, crab, shrimp), insects (e.g., butterflies, flies), as well as in the cell walls of certain fungi (e.g., Aspergillus niger, Penicillium chrysogenum), and green algae [35]. Depending on the species, crustacean shells are composed of protein (20-40%), calcium carbonate (20-50%), and chitin (15-40%) [36]. However, not all sources have the same chitin content (Fig. 1). In natural conditions, the deacetylation of chitin is rarely complete, limiting its application in the industry due to its low solubility. For this reason, the deacetylation process is performed, and when it reaches 50%, chitin becomes chitosan characterized by the degree of polymerization, acetylation, and acetylation pattern [37]. Chitosan consists of N-acetyl D-glucosamine and D-glucosamine units connected via β-1,4–glycosidic bonds [38].

This biopolymer has been in the scope of research recently due to its biological and technological properties, which include anti-inflammatory, antitumoral, antioxidant, antihyperglycemic, antimicrobial, antifungal, wound healing, and mucoadhesive properties [39]. However, these capabilities depend on the physicochemical characteristics, mainly related to the deacetylation degree (DAD) and molecular weight. In contrast, its charge will depend on the DAD of chitosan and the pH of the media. These properties also have a strong effect on the polymer's solubility. Chitosan with high molecular weight is only soluble in acidic solutions [26], showing less bioactivities than chitosan with low molecular weight. The same occurs with chitosan with high DAD, which has better solubility and stronger biological effects [40]. Chemical and physical changes can be made to chitosan to increase its solubility and produce derivatives that can be used for various purposes by taking advantage of its biological activity [22].

Crustacean shell waste is the primary source of chitin and, thus, chitosan. Its extraction usually involves a pre-treatment of the biomass followed by demineralization, deproteination, discoloration, and deacetylation of chitin to finally obtain chitosan [14]. This process can be performed through chemical and biological methodologies [34,41]. Chemical methods are the most employed at the commercial scale, while the biological method is mainly used at the laboratory scale. However, each method has its advantages and limitations, and its application will depend on the approach to be pursued [35,42].

### Main alternatives for chitin extraction

The extraction of chitin can be performed mainly through two methods: chemical or biological extraction [38,43,44]. Some stages can be followed depending on the source used to obtain chitin. For example, when extracted from exoskeletons and shells, the usual process consists of demineralization, deproteination, and decolorization. Instead, when the source of chitin is fungi, stages like demineralization and decolorization are not necessary [17]. The pretreatment for the chemical extraction of chitin starts with washing the crustacean waste with tap water until there are no more residues of crustaceans’ flesh, followed by a wash with distilled water, drying, and grinding (Fig. 1).

### Chemical extraction

The chemical method comprises three main stages: demineralization, deproteination, and decolorization. The demineralization process, also called decalcification, is usually performed with hydrochloric acid (HCl), but other inorganic acids (nitric acid - HNO_3_, sulfuric acid - H_2_SO_4_) and organic acids (acetic acid - CH_3_COOH, formic acid -HCOOH) can be used (Fig. 1). This stage aims to remove mineral constituents from inorganic matter and obtain a soluble salt [34]. HCl is the preferred acid for large-scale industrial processes due to its cost efficiency and high processing speed, making it suitable for most crustacean shells with high calcium carbonate content. HNO_3_ is effective but more expensive and is selected less frequently due to its environmental impact, although it can be useful when additional oxidation of organic material is needed. H_2_SO_4_ is suitable for performing demineralization and deproteinization in the same stage but can degrade chitin quality [45]. Inorganic acids enable the obtention of chitin with low mineral content (∼1.30%). For small to medium-scale operations prioritizing environmental friendliness, CH_3_COOH and HCOOH are ideal, providing gentle demineralization with minimal environmental harm. However, it has been reported that chitin obtained with CH_3_COOH has a higher mineral content (10.45%) compared to the use of HCl (1.24%) [46]. The selection of acid type will depend on several factors such as processing efficiency, cost, environmental impact, and the desired quality of the final product. After this process, the crustacean wastes are filtered under a vacuum, washed with tap water until the pH becomes neutral, and dried (60 °C for 1-48 h until constant weight) [24,25,42].

After demineralization, the process of deproteination takes place using an alkali treatment. The demineralized shells are treated with NaOH, constantly stirring for 1-72 h at 60-160 °C. Other alkaline solutions such as Ca(OH)_2_, CaHSO_3_, KOH, K_2_CO_3_, Na_2_CO_3_, NaHCO_3_, Na_2_SO_3_, NaHSO_3_, Na_3_PO_4_, and Na_2_S can also be employed for this stage (Fig. 1). Following this treatment, the shells are vacuum-filtered once more and then cleaned with tap water until a neutral pH is achieved. Consequently, the removal of pigments can be performed with acetone or an organic solvent for 10 min at 20-65 °C, followed by drying for 2 h at room temperature. The resulting chitin is washed with tap water followed by deionized water. After this, it is filtered and dried at 60 °C for 24 h to obtain chitin (Fig. 1) [14,38,42]. Before deacetylation, the decolorization process can be performed to remove any pigments on the chitin. This process is performed by immersing the sample in a hydrogen peroxide solution (30%, v/v) with a ratio of 1:20 (w/v) at a temperature of 90 °C for 45 min. Finally, the sample is washed to achieve a neutral pH and is subsequently dried in an oven at 60 °C for 24 h [47]. Other agents such as acetone, hypochlorite, and potassium permanganate can also be employed during this stage [42].

Considering the chemical process most used for the extraction of chitin from shrimp shells (Parapenaeus longirostris), the next stoichiometric ratio was estimated using the data from the proximal analyses of the source (carbonates 20 g, protein 40 g, and chitin g 24 per 100 g of shells power) [48]. The calculation was performed following the protocol established by Trung et al. [49]. The demineralization process is determined by Eq. 1.

(1) $$CaCO_{3}+2\text{HCl}\to CaCl_{2}+H_{2}O+CO_{2}↑$$

Considering the molecular weight of CaCO_3_ (100 g/mol), for each 100 g of sample, 0.40 mol of HCl is required. In the established protocol, the HCl was used at 0.80 M at a ratio 1:3.5 (w/v), therefore 350 mL of HCl should be required to completely remove the carbonates in this sample. On the other hand, the deproteinization is governed by Eq. 2.

(2) Protein+NaoH→SolubleProtein−NaComplex

Considering an average protein weight of 110 g/mol, 0.364 mol of NaOH is needed for protein removal, which means a volume of 250 mL of NaOH at 0.75 M considering a ratio of 1:2.5 (w/v). In most studies, proximal analyses of the raw material are not performed, and extraction conditions are set based on other reports without considering the reaction stoichiometry. Therefore, to achieve optimal results, the initial phase should involve analyzing the composition of the material and then determining the appropriate concentration and volume of the reactive substance based on the reactions.

### Biological extraction

Enzymatic reactions and fermentation are used in biological extraction to create acidic and alkaline compounds from enzymatic metabolites and fermentation [43]. Single-step fermentation has been employed for chitin extraction, utilizing lactic acid-producing bacteria such as Lactobacillus acidophilus, Lactococcus lactis, Lactobacillus plantarum, Lactobacillus rhamnoides, Lactobacillus casei, Bacillus amyloliquefaciens, Bacillus licheniformis, and Brevibacillus parabrevis [[50], [51], [52]], which can also produce proteases. Microroganisms like Exiguobacterium profundum Serratia marcescens, Bacillus amyloliquefaciens, and Bacillus sp. have great importance since they produce proteinase of high activity [51,53]. Therefore, deproteinizing and demineralizing processes can be performed simultaneously [52]. The mixture of shell waste powder and culture medium is placed in a shaker incubator (150 rpm) at 30-37 °C for 48-96 h. To obtain dried chitin, the fermented sample is filtered, cleaned with deionized water, and dried at 50–60°C for 3-4 h (Fig. 1) [[51], [52], [53]]. To enhance the result of these processes, a two-step fermentation using Lactobacillus acidophilus and E. profundum is a viable alternative (Table 1) [53]. In biological extraction, culture conditions such as temperature, pH, cultured time, culture medium, carbon source, inoculum level, and others will vary depending on the microorganisms employed to extract the chitin, significantly influencing its properties [51].

**Table 1:** Methods and conditions used for chitin extraction from different sources.

Extraction method	Source	Methodology/Extraction conditions	Chitin yield (%)	Demineralization (DM, %)	Deproteination (DP, %)	References
Chemical	Shrimp shells (Litopenaeus vannamei)	DM: 0.80 M HCl ratio 1:3.5 (w/v) at 28–32°C for 12 h. DP: 0.75 M NaOH ratio 1:2.5 (w/v) at 28–32°C for 24 h.	-	99	99	[49]
	Shrimp shells of Penaeus monodon	DM: 2 M HCl at ratio 1:20 sample to solvent at 25°C. DP: 2 M NaOH at ratio 1:15 (w/v) at 55°C for over-night.	21.88	-	-	[59]
	American lobster (Homarus americanus)		20.22	-	-	
	Crab (Callinectes amnicola)	DM: 3.25 M HCl, ratio 1:10 (w/v) at 28°C for 18.55 h, at 100 rpm. DP: 2.39 M NaOH ratio 1:15 (w/v) at 70°C for 2 h, at 100 rpm.	19.36	-	-	[60]
	Shrimp (Penaeus notialis) shells	DM: 3.25 M HCl at ratio 1:10 (w/v) at 28°C for 19.03 h, 100 rpm. DP: 2.43 M of NaOH ratio 1:15 (w/v) at 70°C for 2.03 h, 100 rpm.	26.08	-	-	
	Shrimp shells (Omani)	DM: 3% HCl ratio 1:10 (w/v) at 25°C for 1 h. DP: 50% NaOH (w/v) at 110°C for 3 h.	53.313	56.2	53.13	[61]
	Smooth shirmp shells (L. vannamei)	DP: 2 M NaOH, ratio of 1:16 (w/v) at 25°C for 24 h. DM: 1 M HCl ratio of 1:16 (w/v) at 25°C for 24 h.	30.09 ± 0.92	-	-	[62]
	Rought shirmp shells (L. vannamei)		26.80 ± 1.05	-	-	
	Lobster (Cherax quadricarinatus) shell	DM: 1.5 M HCl at a ratio of 1:15 (w/v) sample to solvent, at 60–70°C for 4 h and stirring at 50 rpm. DP: 3.5% NaOH at ratio of 1:10 (w/v) sample to solvent, at 60–70°C for 4 h at 50 rpm.	12.99	-	-	[63]
	Blue swimmer (Portunus pelagicus) crab shell	DM: 40 g of the sample was treated with 2 M HCl solution for 24 h at 80°C. DP: The sample was added in 2 M NaOH solution at 110 °C for 20 h at ratio of 1:10 (w/v) Decolorization: Acetone.	20.34 ± 0.72	-	-	[64]
		Pretreatment: crab shells added in 0.5 M acetic acid for 24 h, at ambient temperature. DM: samples were mixed with 25% citric acid for 24 h, at 25°C, washing and drying at ambient temperature. DP: samples were mixed with 5% NaOH for 48 h at 25°C, washing the excess alkali. DC: crude chitin flakes were mixed with 100% n-butanol for 24 h (room temperature), drying the flakes well until all moisture is removed at 60°C.	32.52 ± 0.68	-	-	
Chemical and emerging technologies	Shrimp shells	DM: 0.6 M HCl at a ratio 1:11 (w/v) sample to solvent for 3 h at 30 °C and 300 rpm. DP: High frequency ultrasound in deionized water for 40 min.	57.46	-	-	[15]
Biological and chemical	Shrimp shells	One step fermentation: 100 mL of liquid medium with 3% (w/v) shrimp waste, 0.05% MgSO_4_·7H_2_O, 0.1% K_2_HPO_4_. Incubated at 37°C, 150 rpm for 96 h. DP: 4% (w/v) NaOH at 100°C for 1 h; 1.25 M HCl at 25°C for 1 h.	23.23	21.3	95	[50]
Biological	Pacific white leg shrimps (Litopenaeus vannamei)	Single step fermentation: 10 g of sample, glucose 15% (w/w), Lactobacillus acidophilus FTDC3871 5% (∼10^9^ CFU/mL, v/w), and water (100 mL) for 72 h at 37°C	58.46	90.8	76	[52]
	Shrimp shells	Two-step fermentation: Lactobacillus acidophilus followed of Exiguobacterium profundum each one at 10^8^–10^9^ CFU/mL, 140 rpm, 25°C for 120 h	16.32	95 ± 3	85.9 ± 1.2	[53]
	Shrimp shells (Litopenaeus vannamei)	Two-step fermentation: 5 g of powder shrimp shells were added to 100 mL of water with 5% glucose and 4% Lactobacillus rhamnoides (10^9^ CFU/mL), pH 6.5, at 37°C for 48 h, 5% glucose. Bacillus amyloliquefaciens BA01 6% (10^9^ CFU/mL), pH 6.5, at 37°C for 84 h, and 4% glucose.	19.6	97.5	96.8	[51]
	Shirmp shells	Two step fermentation: 100 g/L of shrimp shell and 50 g/L glucose, and Lactobacillus delbrueckii for 24 h, then a pulse of 35 g/L glucose was added, and at 48 h an inoculum of Bifidobacterium lactis was added. These cultures were incubated at 40°C and 200 rpm for 72 h, the pH was maintained at 6.0.	–	98.63	88	[65]
		5% Lacticaseibacillus paracasei at solid-liquid ratio of 1:3, in 15% glucose, at 37°C for 48 h.	-	61.49	46.38	[66]
Biological and emerging technology	Crab shells	5% Lacticaseibacillus paracasei at solid-liquid ratio of 1:3, in 15% glucose, at 37°C with low-intensity ultrasound (0.167 W/cm2, 40 kHz) for 10 min at 8 h intervals for 48 h	-	71.77	59.50	

DM: Demineralization; DP: Deproteinization; DC: Decolorization process

Enzymes, particularly proteases, play a vital role in biological extraction, promoting the extraction of chitin with higher molecular weight than those extracted chemically [54]. Several studies have focused on evaluating the demineralization process based on chemical reactions followed by enzymatic deproteinization. However, it is important to consider that the different inter- e intramolecular interactions of chitin and chitosan result in the obtention of α- or β-chitosan. These structural modifications affect their susceptibility to enzyme treatments, α-chitosan is less reactive to cellulase hydrolysis than β-chitosan due to its high crystallinity. As a result, the deacetylation degree and molecular weight (75% DDA/31 kDa and 90% DDA/74-76 kDa for α- and β-chitosan, respectively) can vary, influencing their physicochemical and biological properties [55]. Properly selecting enzymes is critical to the polymer's properties [18,54].

#### Emerging technologies for chitin extraction

Recently, techniques based on physical forces have been employed for chitin extraction from fishery wastes. In this regard, the generation of the waves created by the cavitation phenomenon during ultrasound-assisted extraction (UAE) has been coupled to the demineralization process to achieve deproteinization and obtain chitin. Using high-frequency ultrasound for 10 to 40 min enables the extraction of chitin from shrimp shells with yields ranging from 22.77 to 71.53% [15,16,56], which is higher than the typical yields obtained with chemical and biological methods (Table 1). Ultrasonic waves can enhance the extraction process, reducing both extraction time and energy consumption compared to traditional methods. Squid pens (Loligo formosana) ultrasonicated for 41.46 min at an amplitude of 69% and a solid/solvent ratio of 1:18 yielded 34.65% of high-purity chitin (3.49 mg of protein/100 mg sample). Reducing the extraction period required by traditional methods by 7.2 times (traditional yield 38% and 5.1 mg protein/100 mg sample) [16]. Ultrasound waves may break down compounds accelerating chitin degradation, which can be removed during the washing process. Also, the higher yield of chitin obtained by the traditional technique is associated with a higher residual protein content than in chitin extracted by the UAE. Compared to the conventional method, UAE efficiently lowers the protein content of the chitin and shortens the extraction time [15,16]. Moreover, UAE requires optimization because the size of chitosan particles can decrease to 35 μm while the DAD increases from 73 to 100% as processing time increases from 10 to 35 min. Compact and eroded particles have a higher degree of crystallinity, corresponding to the ß-chitosan structure, used in biomedical applications [15]. The evaluation of high-intensity ultrasound (HIU) followed by co-fermentation with Bacillus subtilis has been studied for chitin extraction from shrimp shells. As a result, pre-treating the raw material with HIU at 800 W increased protease activity in the fermentation compared to the untreated shells, reducing the fermentation time from 5 to 4.5 days. Chitin purified from shrimp shells pre-treated with HIU at 800 W exhibited lower molecular weight (11.2 kDa), higher purity (89.8%), and a higher DAA (21.1%) compared to the untreated samples (13.5 kDa, 86.6%, and 18.5%, respectively). This is attributed to the ultrasound effectively removing the protein/CaCO_3_ matrix covering the shrimp shells’ power surface compared to untreated samples [56]. The reduction of protein content in chitin particles increases contact with the solvent during the DDA process, resulting in higher deacetylation values [15,16]. On the other hand, applying ultrasound waves can enhance the fermentative process by stimulating extracellular β-galactosidase, which increases acidity, thus favoring the fermentation, demineralization, and deproteinization processes (Table 1).

Microwave-assisted extraction (MAE) is a rapid and energy-efficient method for chitin and chitosan extraction, utilizing microwave energy to heat the raw material and facilitate extraction [18,57]. MAE can be combined with the use of deep eutectic solvents (DES) to enhance chitin extraction. For instance, a DES-based on choline chloride/lactic acid (1/10) was employed to extract chitin from crayfish shell wastes via MAE (using a solvent-sample ratio of 10/1, at 120 °C for 30 min at 300 W), resulting in an extraction yield of 19.11% with a purity of 97.44% [57]. In addition to the high yield and purity, another important advantage of MAE is its lower energy consumption compared to conventional extraction processes. For example, it has been reported that for chitosan extraction from the mycelium of Rhizopus oryzae NRRL 1526, MAE (300 W for 22 min) consumes only 0.11 kW h of energy and provides a higher yield of chitosan with higher DAD (yield 13.43 ± 0.3%, DAD: 94.6 ± 0.9%), while the conventional process consumes 5 kW h (yield 6.67% ± 0.3%, DAD 90.6 ± 0.5%). This significant reduction in energy consumption can lead to cost savings from 50 to 1.1 cents [17]. The effect of this technology has been assessed for each step of chitosan extraction from Moroccan shrimp shells. Microwave irradiation at 500 W enables the removal of 95% of calcium carbonates from the shells (demineralization) in 8 min using HCl (30%), the deproteinization was performed using NaOH (20%) at 500 W for 8 min (protein removal 96%). Then, the obtained chitin was deacetylated with NaOH (30%) for 12 min at 500 W (DAD: 23.4%). Hence, it is possible to obtain chitosan with low DAD in a short time, with high crystallinity and molecular weight by using MAE as a sustainable method [58], reducing both extraction time and the concentration of needed solvents [17,58]. However, it is important to note that using microwave irradiation may potentially affect the properties of chitin or chitosan, mainly related to a reduction in molecular weight linked to increases in power and reaction time [58]. The proper conditions control during UAE or MAE and its combination with chemical and biological processes presents an alternative to obtaining high yields of high-quality polymers while reducing or avoiding the need for chemical solvents and extraction time. It is important to consider the optimization of the chitin extraction process due to significant variations in yield and purity (mineral and protein content) depending on the source, extraction method, and conditions used during the extraction process (solvent type, solvent-to-sample ratio, extraction time, temperature) (Table 1). Scaling up UAE and MAE for chitosan extraction presents several challenges, such as maintaining uniform energy distribution and managing heat dissipation across large volumes of extraction media. Therefore, the extraction parameters (such as frequency, amplitude, microwave power, solvent type, and treatment time) for large-scale operations require extensive experimentation and fine-tuning. The initial investment and operational costs can be significant due to large-scale design, fabrication, and equipment maintenance. Finally, large-scale US operations can generate noise pollution [[57], [58], [59]].

### Chemical and physical deacetylation of chitin

Decolored chitin can be transformed into chitosan by removing the acetyl group. The traditional method for deacetylation involves exposing the chitin to concentrated alkaline solutions, typically NaOH (0.5 to 2.5 M) [25]. In this process, dried chitin is immersed in the alkaline solution and stirred for 12 h. Subsequently, the mixture is heated at 90-100 °C for another 12 h. Finally, the mixture is filtered, and washed with deionized water, and the resulting chitosan is dried at 60 °C until a constant weight is achieved [47,52,67]. Chemical deacetylation remains the primary method for producing chitosan (Table 2). Otherwise, low-frequency ultrasonic irradiation has been used as a cost-effective alternative for chitin deacetylation at relatively low temperatures (below 70°C) and short reaction times (up to 120 minutes). The results showed that the produced chitosan had a DDA of up to 87.73% under optimal conditions, compared to 66.82% using the conventional thermo-alkaline process. The characterization showed that the chitosan obtained with the pretreatment has the same fingerprint as the commercial polymer. However, a significant breakage of the glycosidic bond in the polymer structure was observed, and the DDA reduced the thermal stability of the obtained product [68]. The breakage of glycosidic bonds results in the cleavage of the polymer chains, thereby reducing the molecular weight of chitosan. Low molecular weight can affect the solubility, viscosity, mechanical properties, film-forming ability, and crystallinity of chitosan [56,69].

**Table 2:** Main conditions for chitosan obtention and deacetylation degree

Source	Chitosan extraction conditions	Chitosan yield (%)	Chitosan DDA (%)	References
Pacific white leg shrimps (Litopenaeus vannamei)	50% NaOH, a ratio of 1:20 (w/v), and incubated in a water bath at 100°C for 12 h	∼52	-	[52]
Shrimp shells	10 mL of crude deacetylases cocktail from Aspergillus niger (35 U/mL) was mixed with 1.0 g crude chitin and 10 mL acetate buffer pH 5.0. Incubation at 40°C and 200 rpm for 24 h. Filtration, washing with tap water, and drying at 60°C for 24 h.	-	74	[65]
Shrimp shells (L. vannamei)	12.5 M NaOH solution (1:5 w/v) at 65°C for 12 h. Wash with demineralized water and dry in sunlight	-	88.76	[49]
Crab (Callinectes amnicola) shells	50% (w/w) NaOH at 85.05°C for 133.64 min	13.29	84.2	[60]
Shrimp (Penaeus notialis) shells	50% (w/w) NaOH at 87.9°C for 145.26 min	16.93	89.73	
Shrimp shells (Omani)	10% NaOH ratio l (1:10 w/v) for 15 min at 121°C	4.94 ± 0.17	-	[61]
Smooth shirmp shells (L. vannamei)	50% NaOH for 24 h at 25°C, washed until pH 7, and drying at 70°C	20.75 ± 0.83	84.08 ± 1.27	[62]
Rought shirmp shells (L. vannamei)		19.34 ± 1.30	80.78 ± 0.79	
Crab shells	Chitin incubation in 3 N HCl at a ratio of 1:5 (w/v). Soaking with NaOH 1.5 N (with a ratio of 1:10, w/v), heating it at 130°C for 3 h. Neutralization with distilled water and drying at a temperature of 30-40°C for 24 h.	6.51 ± 0.22	88.29 ± 0.31	[70]
Green mussel shells		11.60 ± 0.61	48.68 ± 0.44	
Shrimp shells of Penaeus monodon	48% (w/v) NaOH for 4 h at 100°C (ratio 1:20 w/v). Wash with distilled water and dry at 55°C for overnight.	17.25	82	[59]
American lobster (Homarus americanus)		16.68	-	
Lobster (Cherax quadricarinatus) shell	60% NaOH, ratio of 1:20 (w/v) at 100–110°C for 4 h at 50 rpm.	8.93	68.75	[63]
Blue swimmer (Portunus pelagicus) crab shell	50% NaOH (w/v) solution at 150°C for 4 h, at ratio of 1:10 (w/v).	13.79 ± 0.93	79.40 ± 3.35	[64]
	50% NaOH at 120°C for 4 h to obtain crude chitosan, washing and drying. Purification with 2.5% EDTA and 5% SDS for 2 h at 125 rpm overnight at room temperature. Wash and dry at 60°C.	21.78 ± 0.34	82.54 ± 1.73	
Shrimp shells	50% NaOH at ratio 1:4 (w/v) sample to solvent, at 70 °C for 2 h, followed by 115 °C for 2 h at 700 rpm.	11.074	100.00	[15]

### Biological deacetylation of chitin

The enzyme chitin deacetylase (CDA) derived from Mucor rouxii and Bacillus cereus, is responsible for the conversion of chitin into chitosan [65,71]. In this process, the CDA-producing strain is cultivated in an optimized fermentation medium containing chitin, for example at 37 °C, pH 6, and 180 rpm for 48 h for B. cereus. Subsequently, the fermentation broth is centrifuged (5 min at 4°C, 9000 rpm) and the resulting precipitate is the chitosan. The degree of deacetylation depends on the extracellular CDA activity of the strain, which is influenced by factors such as pH, temperature, carbon source, and nitrogen source utilized for the growth of the CDA-producing strain (Table 2) [72]. This method is not widely used, even though recombinant CDAs can be produced using microbial expression systems, such as Escherichia coli, but optimizing the production process and achieving high yields can be complex. The production of CDAs with specific properties, such as tailored acetylation patterns is challenging due to the need for expensive equipment, purification procedures, quality control measures, and sensitivity to environmental conditions, such as temperature and pH. These factors contribute to the overall cost of production, making CDAs less economically viable for commercial use [73,74]. Each method presents advantages and disadvantages concerning the properties of the obtained polymer and their feasibility for large-scale or industrial implementation (Table 3).

**Table 3:** Comparison of chemical and biological chitosan extraction methods.

Extraction method	Treatment	Advantages	Disadvantages	
Chemical	Demineralization: concentrated acids (HCl). Deproteination: alkaline solutions (NaOH)	-Industrial production -Low operation costs -Higher yield -Fast reaction time -Simple extraction -Chitosan with medium to low molecular weight and high deacetylation degree	-Environmentally harmful -Use of toxic and corrosive chemicals -High energy consumption -Production of acids and alkaline wastes	[49,[60], [61], [62]]

Biological	Demineralization: lactic acid producing bacteria. Deproteination: proteases producing bacteria.	-Environmentally friendly -Mild reaction conditions -Chitosan with high molecular weight -High quality chitin	-Long fermentation times -High operation costs -Inability to eliminate final residues -Slow reaction time -Complicated extraction -Use of specific microbial strains	[50,52,65]

Emerging technologies	Demineralization and deproteinization process can be performed in one step using enabling the use of deep eutectic solvents	-Environmentally friendly -Reduction of extraction time -Chitosan with high purity -Easy to scale up	-Implementation cost.	[57,64]

#### Chitosan modification: alternatives to improve its properties

Chitosan possesses numerous functional features but is limited by its poor solubility. A solution to this challenge is modification, which imparts chemical and biological advantages over unmodified chitosan [75]. Typically, modifications can be performed through chemical, physical, or a combination of both processes. Physical modifications involve the conversion and blending of chitosan [19], while chemical modifications entail alterations to the primary and secondary hydroxyl and amino functional groups [76]. Chitosan can undergo various modifications and functionalization, such as addition/coupling and cross-linking with other molecules, enabling it to exhibit high selectivity [44]. Some chitosan modifications that have been studied include graft copolymerization, quaternization, carboxymethylation, alkylation, thiolation, acylation, hydroxylation, phosphorylation, phthaloylation, sulfonation, methylation, nitration, xanthation, and cross-linking, among others [42,44,75]. Among these, graft copolymerization stands out as the most widely used method of chitosan chemical modification [28]. By covalently bonding small molecules, mainly hydroxyl groups and the free amino groups, to the chitosan chain using a chemical redox initiator (such as ceric ammonium nitrate, ammonium, and potassium persulfate), this technique facilitates the production of functional derivatives. In addition, this modification can be achieved through grafting initiated by free radicals, gamma radiation, microwave irradiation, and enzymatic methods [[19], [20], [21],^29^,^75^]. Grafted chitosan demonstrates improved stability, water solubility, antioxidant, antibacterial, and antifungal properties, while also enhancing chelating properties and adsorption capacity. Moreover, it can introduce new properties such as biodegradability, mucoadhesiveness, and biocompatibility [29]. The improvement of chitosan's properties by graft copolymerization is linked to the introduction of functional groups such as hydroxyl, carboxyl, amine, and sulfonate groups. These functional groups provide additional sites for interaction and binding, thus enhancing the overall performance and versatility of the modified chitosan [20]. The addition of these functional groups makes chitosan an alternative for the design of controlled-release systems, which is crucial for preserving the bioactivity of drugs and bioactive compounds [20,77].

Chitosan quaternization involves methylating or grafting the quaternary-ammonium group of the polymer [78]. This occurs when the amino terminals of chitosan are replaced with quaternary terminals or through the addition of functional cationic groups. Quaternary ammonium chitosan derivatives can be obtained via chemical reactions with methyl iodide, sodium iodide, or sodium hydroxide. Quaternary ammonium groups improve chitosan's solubility over a wide pH range and enhance its antimicrobial properties, providing mucoadhesiveness [79]. Quaternization increases the hardness of chitosan [56], making it suitable for applications in film and coating production and as a carrier for active ingredients, thereby enhancing the stability and quality of food products [78]. In addition, quaternary ammonium groups make chitosan particularly effective for antimicrobial coatings on medical devices and surfaces [20,80]. On the other hand, carboxyalkyl chitosan is obtained by introducing acidic groups to the amino groups of chitosan, resulting in an amphoteric electrolyte mixture with both cationic and anionic charges [21]. Carboxymethylation increases chitosan’ solubility across different pH levels by adding carboxymethyl groups. The modified polymer enables the obtention of viscous solutions, enhancing film-forming ability and bioadhesive properties, making it ideal for wound dressing applications that require moisture retention and adherence to wound sites [76,81]. Alkylated chitosan derivatives are produced by reductive amination of chitosan's amino groups using aliphatic aldehydes with alkyl groups, which are then attached to the amino group of chitosan [82]. The solubility of alkylated chitosan depends on the length of the alkyl group; short chains improve solubility, while long chains may reduce it, but overall, they enhance the thermal stability of the polymer. This modification results in alkyl chitosan with amphiphilic character and slight cationic charge under neutral pH conditions, thereby enhancing properties such as solubility, antimicrobial activity, chelating ability, adsorption capacity, and amphiphilicity [21,83]. The added hydrophobic alkyl groups promote the interaction of chitosan with hydrophobic drugs, improving their solubility and stability in delivery systems. This modification is particularly advantageous for delivering hydrophobic pharmaceuticals [23,76]. The alkyl chain length and the degree of substitution have been optimized to obtain a chitosan cryogel with improved mechanical properties compared to the unmodified polymer. As a result, octanal (C8) was found to be the optimal cryogel for mechanical performance, whereas dodecanal (C12) was optimal for the adsorption/release of hydrophobic drugs. These results indicate that cryogels based on hydrophobically modified chitosan have significant potential as biomedical materials [23].

#### Applications in the industry

**Fig. 2:**
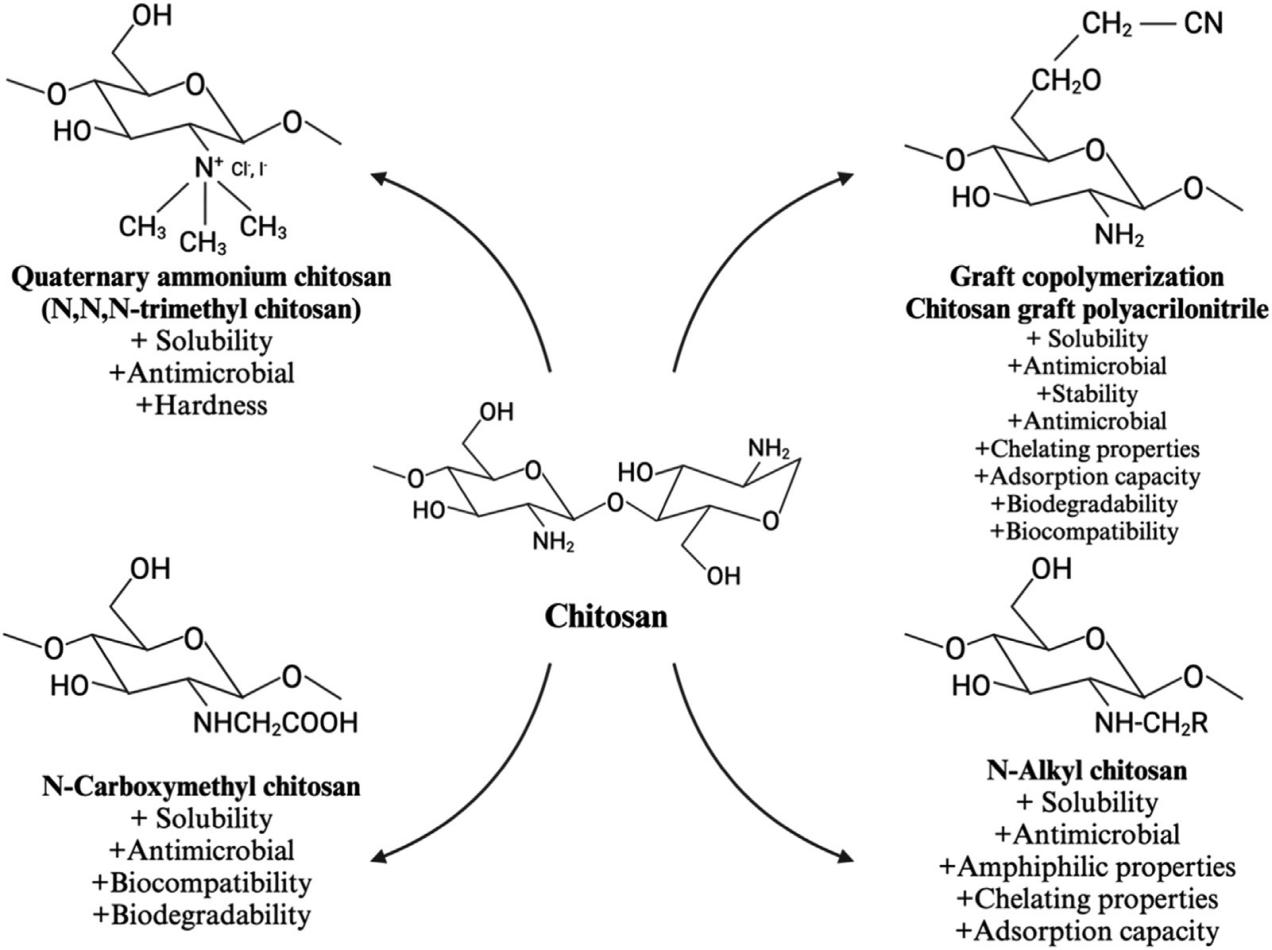
Chitosan modifications and its enhanced features.

Chitosan and its derivatives have demonstrated applications across various sectors, including medicine, agriculture, plastics, paper, cosmetics, energy, and food [25]. Their broad applicability is attributed to their antibacterial, antifungal, antioxidant, anti-inflammatory, anti-cancer, fat-binding, film-forming, and chelating properties [34]. Within the food industry, chitosan and its modifications find use in food packaging, preservation, supplements, and encapsulation systems (Fig. 2). In food packaging, chitosan's antimicrobial activity is utilized to create self-preserving packaging, thereby maintaining the nutritional quality of food [12]. Acting as an environmentally friendly packaging material, chitosan also serves as active food packaging with barrier properties that enhance gas exchange and protect the aroma of food products [75]. This biopolymer is to preserve fruits, vegetables, and meat by delaying the growth of spoilage microorganisms and maintaining food quality [29]. Water-soluble chitosan variants such as alkyl, quaternary, and carboxymethyl chitosan serve as food packaging additives, enhancing barrier properties [84]. Grafted chitosan, through the grafting of acrylic acid, acrylonitrile, acrylamide, dicyandiamide, phenolic acids, and other grafting chains, has been utilized in food packaging to reduce microbial infection and preserve food products [29]. For instance, gallic acid grafted with carboxymethyl chitosan has shown a prebiotic effect and improved digestive stability of the polymer [85]. Quaternary chitosan has been employed to enhance microbial, hydrophobic, and mechanical properties of food packaging films as well [86]. In addition, alkylated quaternary ammonium chitosan has been employed to reduce microbial contamination in leafy vegetables [87]. Chitosan derivatives, particularly those with an 83% deacetylation degree (edible), have been used as dietary food additives and supplements for controlling obesity. Protonation of the amino group in chitosan at acidic pH (such as the pH in the human stomach) provides the molecule a strong positive charge, which attracts negatively charged particles, including oils and fat-containing phospholipids. This results in micelles and fat emulsions that remain indigestible by the human body, making it a viable product for weight loss [16,88]. Furthermore, it has also been utilized for beverage preservation, clarification, and encapsulation of bioactive compounds [75]. It is important to note that the use of chitosan as a weight supplement is still a controversial topic. Studies involving humans have assessed its efficacy for 12 to 56 days without observing the toxicity or apparent harm after oral chitosan treatment [89,90]. A long-term study in rats fed with chitosan (1,500 and 5,200 mg chitosan/kg body weight per day for males and 1,800 and 6,000 mg/kg per day for females) for 6 months showed a significant decrease in serum vitamin A, serum and hepatic vitamin E, and increased levels of serum 1.25 (OH)_2_ vitamin D. High chitosan consumption lowered the proportion of fat that was digested, increased the weight and moisture of the feces, and decreased the levels of phosphorus, cholesterol, and triglycerides. Significant liver weight and histology alterations were also observed in female rats exposed to 6,000 mg of chitosan/kg per day [91]. Given the lack of long-term human studies and the significant effects observed in rats, using chitosan as a weight supplement in humans should be approached with caution. Further long-term studies in humans are needed to gain a better understanding of safety and efficacy. Regularly monitoring liposoluble vitamin levels is recommended for individuals who choose to consume chitosan supplements to avoid potential deficiencies.

Chitosan and its derivatives have potential applications in biomedical, pharmaceutical, biotechnological, agricultural, cosmetic, paper, textile, and water treatment industries [25,26,76]. In the cosmetic industry, chitosan serves as an antimicrobial, antioxidant, carrier for active ingredients, and film-forming agent. Its use in the biomedical sector is extensive due to its biocompatibility, biodegradability, and non-toxic nature [34]. Chitosan-based bionanomaterials have been employed for tissue regeneration and developing drug/gene delivery systems [37]. In wastewater treatment, chitosan can remove fats, heavy metals, and dyes. In the pharmaceutical field, it can act as a carrier for drug administration [12]. Chitosan with a high deacetylation degree is commonly applied for wound healing, drug delivery, dietary ingredients, food preservatives, wastewater treatment, molecule imprinting, metal reduction, and textiles. Chitosan with a deacetylation degree exceeding 85% is employed for gene delivery, nanoparticle stabilization, and emulsification. Low molecular weight chitosan is used for biological applications due to its antioxidant, antimicrobial, and antiviral properties, particularly as part of a food-protectant system [[92], [93], [94]]. Chitosan with a high molar mass is suitable for drug delivery, tissue engineering, enzyme immobilization, and wound healing [[25], [26], [27],^71^].

**Fig. 3:**
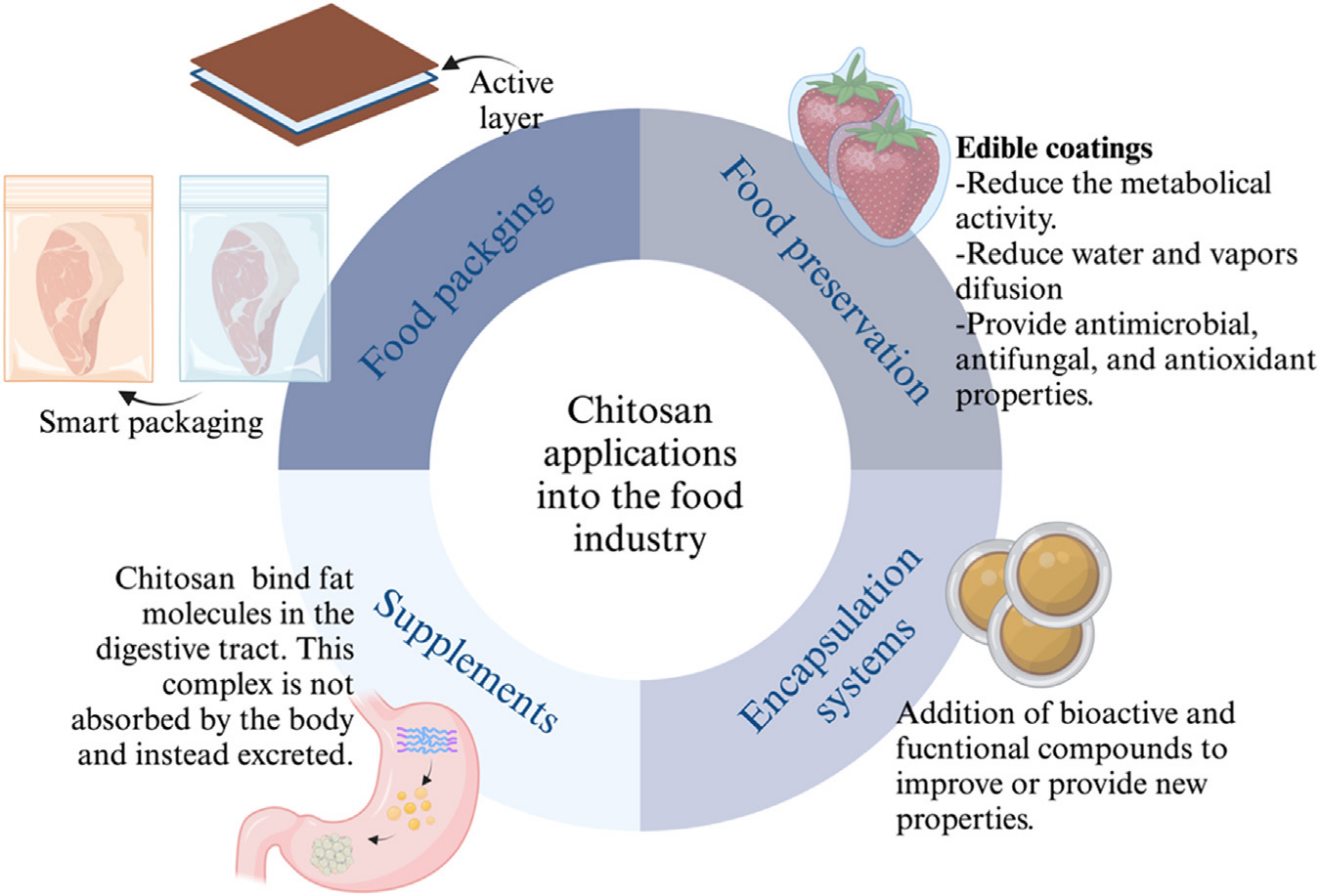
Main applications of chitosan in the food industry.

Implementing chitosan-based products on an industrial scale comes with several challenges. The isolation and purification processes of chitosan from its sources need to be optimized and scaled up for industrial production. The implementation of the Safe-by-Design approach can be an important tool in this task [95]. When comparing chitosan with synthetic plastics, this biopolymer has lower performance characteristics, such as a lower water vapor barrier, thermal stability, and mechanical properties. This involves addressing challenges related to energy consumption, wastewater treatment, and cost-effectiveness [69]. In addition, a critical issue with the use of chitosan in food packing is the limited number of studies evaluating the interaction between chitosan and the packed or coated foodstuffs. Regarding chitosan applications in wastewater treatment, the study of modifications that enhance its resistance to acidic environments and mechanical weakness should be evaluated at the laboratory scale [35]. Barriers to the entrance of chitosan in industrial sectors include scale-up cost, product differentiation, and regulatory barriers. Regulatory agencies assess chitosan and its derivatives based on their intended use, rather than providing a general definition of chitosan as Generally Recognized as Safe (GRAS). Depending on the target application and industry sector, different organizations are involved in the evaluation and certification of the product. For the food and pharmaceutical industries, the Food and Drug Administration regulates the process [96]. On the other hand, if the intended use is for developing biopesticides and seed decontamination, the Environmental Protection Agency is the responsible institution [97,98]. Depending on the application and target market, local, national, and international institutions can be involved in the large-scale implementation of chitosan, (Fig. 3).

## Conclusion

The fishery industry generates waste alongside consumption, including bones, scales, and shells. Shells from marine species are rich in chitin, traditionally obtained through chemical processes involving inorganic acids and alkalis. To reduce the use of inorganic solvents, fermentation has been proposed as an alternative for obtaining high-quality polymers. However, the biotechnological process can increase extraction times by 3 to 5 times compared to chemical extraction. The combination of ultrasound or microwave-assisted extraction with chemical and biological processes seems to be the most effective approach for addressing issues related to harsh chemical compounds and long extraction periods without compromising yield and polymer quality. The quality and quantity of the obtained chitin depend on several factors, which should be carefully selected based on the polymer's application. The deacetylation of chitin produces chitosan, which is of major interest for its outstanding properties. Chitosan can be further modified to obtain derivatives with an expanded range of applications. Graft copolymerization, quaternization, carboxylation, and alkylation are among the chitosan modifications explored to enhance solubility, bioactivity, adsorption, stability, and other properties. The versatility of modified chitosan enables its application across various sectors including food, medical, agricultural, and biotechnological, making it a valuable biopolymer with numerous benefits. Further studies should focus on the evaluation of chitosan extraction by combining biotechnological tools and emerging technologies, optimizing conditions based on the source, and considering the stoichiometric ratio.

## Ethics statements

Not applicable.

## CRediT authorship contribution statement

**Maricarmen Iñiguez-Moreno:** Conceptualization, Writing – original draft, Visualization. **Berenice Santiesteban-Romero:** Conceptualization, Writing – original draft, Visualization. **Elda M. Melchor-Martínez:** Writing – review & editing. **Roberto Parra-Saldívar:** Supervision. **Reyna Berenice González-González:** Supervision, Writing – review & editing.

## Declaration of competing interest

The authors declare that they have no known competing financial interests or personal relationships that could have appeared to influence the work reported in this paper.

## Data Availability

No data was used for the research described in the article.
